# The Estimation of Knee Medial Force with Substitution Parameters during Walking and Turning †

**DOI:** 10.3390/s24175595

**Published:** 2024-08-29

**Authors:** Shizhong Liu, Ziyao Wang, Jingwen Chen, Rui Xu, Dong Ming

**Affiliations:** 1 Academy of Medical Engineering and Translational Medicine, Tianjin University, Tianjin 300072, China; liu_rehab@tju.edu.cn (S.L.); wangziyao@tju.edu.cn (Z.W.); jingwen1013@tju.edu.cn (J.C.); richardming@tju.edu.cn (D.M.); 2 Department of Rehabilitation Medicine, Tianjin Medical University General Hospital, Tianjin 300052, China

**Keywords:** knee adduction moment, knee flexion moment, knee medial force, turning

## Abstract

Purpose: Knee adduction, flexion moment, and adduction angle are often used as surrogate parameters of knee medial force. To verify whether these parameters are suitable as surrogates under different walking states, we investigated the correlation between knee medial loading with the surrogates during walking and turning. Methods: Sixteen healthy subjects were recruited to complete straight walk (SW), step turn (ST), and crossover turn (CT). Knee joint moments were obtained using inverse dynamics, and knee medial force was computed using a previously validated musculoskeletal model, *Freebody*. Linear regression was used to predict the peak of knee medial force with the peaks of the surrogate parameters and walking speed. Results: There was no significant difference in walking speed among these three tasks. The peak knee adduction moment (pKAM) was a significant predictor of the peak knee medial force (pKMF) for SW, ST, and CT (*p* < 0.001), while the peak knee flexion moment (pKFM) was only a significant predictor of the pKMF for SW (*p* = 0.034). The statistical analysis showed that the pKMF increased, while the pKFM and the peak knee adduction angle (pKAA) decreased significantly during CT compared to those of SW and ST (*p* < 0.001). The correlation analysis indicated that the knee parameters during SW and ST were quite similar. Conclusions: This study investigated the relationship between knee medial force and some surrogate parameters during walking and turning. KAM was still the best surrogate parameter for SW, ST, and CT. It is necessary to consider the type of movement when comparing the surrogate predictors of knee medial force, as the prediction equations differ significantly among movement types.

## 1. Introduction

As walking and turning are common activities in human daily lives, the knee loading of the countless walking steps is important to the health of the knee [1,2]. Knee medial force (KMF), the force acting at the medial compartment of the knee, have attracted attention as medial knee osteoarthritis (OA) is much more common than lateral [3]. Therefore, the ability to estimate the knee medial loading in individuals would be valuable for identifying the risks of knee degeneration [4,5,6] and the treatment of knee OA [7,8].

The most accurate method for determining the knee medial loading would be to measure the forces in vivo using knee force sensors, but it is only possible for people who have total knee replacements, which limits the application of this measurement [9]. The musculoskeletal models are commonly used to estimate muscle forces and, subsequently, to estimate the internal knee loading non-invasively as a compensation of the in vivo measurement [10,11,12]. Recent musculoskeletal models have been developed to study complex knee biomechanics [13].

Although these models provided an approximate estimation of knee loading, they had to account for complicated information (such as anatomical information and location of each muscle) and calculation (such as static optimization or muscle modelling). For this reason, many relevant researchers could not use these models properly or correctly, and some tried to use surrogate parameters to briefly estimate knee medial loads. Schipplein et al. first proposed that the external knee adduction moment (KAM) is the primary determinant of knee medial loads [14]. Zhao et al. investigated the relationship between the KAM and in vivo knee medial contact force and found that the KAM is highly correlated with internal knee medial loads [15]. While the KAM is commonly used to represent the KMF, some researchers proposed more precise approaches. However, Pieri et al. found the KAM may not adequately describe compartmental load magnitude or changes induced by interventions at the compartment level [16]. Additionally, Walter et al. investigated the relationship between the altered knee medial force generated via gait modification and the corresponding KAM and knee flexion moment (KFM), and found both peaks of medial contact force were best predicted by a combination of the peak KAM and peak absolute KFM [17]. Zeighami et al. also suggested the KAM and KFM remained the best predictors of the medial and lateral knee forces [18]. Similar conclusions were also drawn in studies [19,20,21]. In addition, an informed evaluation of the knee medial loads based on linear regression showed that the KAM and KFM were both significant predictors of the KMF during normal gait [22]. Furthermore, some other researchers suggested that the internal load partitioning is dictated mainly by the knee adduction angle (KAA). Marouane et al. concluded that the KAA should be the primary marker of knee joint load partitioning [23]. Therefore, the KAM, KFM, and KAA have been reported to indicate the alterations of knee medial loads during level straight walking, and the linear model becomes more accurate in predicting the medial knee joint contact force [21].

Walking, however, involves not only going straight but also step turns and crossover turns [24]. As a recent relevant study [25] showed, findings from simple straight walking cannot be generalized to other gait states. Valente, G et al. [26] had pointed out that different task states may have a greater impact on the knee contact locations than the knee joint arrangement. Therefore, the prediction of the KMF of level walking should consider both straight walking and turning states. However, we could not find any study that combined the KAM, KFM, and KAA to evaluate the pKMF during straight walking and turning.

Therefore, the present study aimed to investigate the differences in the KMF, KAM, KFM, and KAA between walking and turning. Additionally, we evaluated the KMF during straight walk (SW), step turn (ST), and crossover turn (CT). First, peaks of the KMF, KAM, KFM, and KAA (pKMF, pKAM, pKFM, and pKAA) were calculated for SW, ST, and CT. Second, linear regression models were built to explore the evaluations of the pKMF for different tasks. Third, the predictors of different tasks and their interclass correlation coefficients were statistically compared. It was hypothesized that the knee biomechanical parameters varied across different walking tasks, and the estimation of the KMF may be influenced by the tasks. The findings may help understand the predictors of knee medial loading for different walking tasks and the biomechanical characteristics of these tasks. Furthermore, this study may improve the accuracy of knee medial loading estimation based on external knee moments.

## 2. Materials and Methods

### 2.1. Participants

A total of 16 healthy students from Tianjin University (7 females and 9 males; height: 1.71 ± 0.10 m; mass: 58.83 ± 12.59 kg; age: 23.5 ± 2.8 years) were recruited for participation in the experiment protocol. The experiment was approved by the ethics committee of Tianjin University, and all the participants gave written informed consent before experiments.

### 2.2. Experimental Protocols

**Figure 1:**
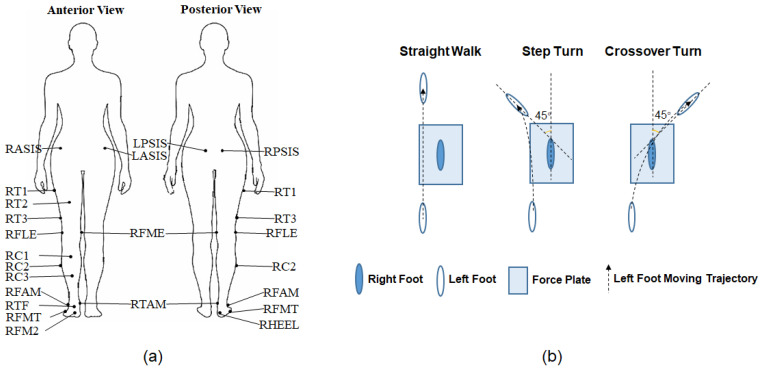
Experimental setup. (**a**) Markers’ attachment. (**b**) Experimental tasks.

The experimental setup for data collection comprised a set of ten Vicon optoelectronic cameras (T20S, Vicon Motion System Ltd., Oxford, UK) and an AMTI force plate (BP400600, Advanced Mechanical Technology Inc., Watertown, MA, USA). Eighteen reflective markers were used in this study, as shown in Figure 1a: RASIS, LASIS (right and left anterior superior iliac spine); RPSIS, LPSIS (right and left posterior superior iliac spine); RT1, RT2, RT3 (cluster markers on the thigh); RFLE, RFME (lateral and medial femoral epicondyle); RC1, RC2, RC3 (cluster markers on the shank); RFAM, RTAM (apex of the lateral and medial malleolus); RHEEL (calcaneus); RFMT (tuberosity of the fifth metatarsal); RFM2 (head of the second metatarsal); and TF (the center of the acrotarsium). Each subject was required to complete one static trial, where the subject stood motionless in the neutral position for 1 s and performed three dynamic tasks with different walking directions (shown in Figure 1b): forward (SW), left (ST), and right (CT).

During the dynamic tasks, subjects walked naturally along the walkway, taking several steps before and after the right foot fully contacted the force plate. For step and crossover turning, the subjects were instructed to turn 45° from the original progression direction. Practices were taken before each task to guarantee the smooth completion. The 3D coordinates of 18 markers, ground reaction force (GRF), and center of pressure (COP) were recorded during dynamic tasks, and only marker positions were recorded for the static trial. The sampling frequency of the motion and kinetic data were 100 Hz and 1000 Hz, respectively. Each dynamic task consisted of six trials of data recording. Four trials with uninterrupted marker trajectories were selected for data processing.

### 2.3. Data Processing

The motion data were first labelled in Vicon Nexus (version 1.85) and then imported into Matlab (R2012b, The MathWorks Inc., Natick, MA, USA) along with the kinetic data. The motion data were filtered using a 4th-order Butterworth zero-phase digital filter with a low-pass cut-off frequency of 6 Hz [27], while the kinetic data were low-pass filtered at 15 Hz [19] and downsampled to match the motion data. The walking speed (WS) was calculated according to the marker’s position at the right heel for all the subjects and tasks.

$$WS=\frac{\sqrt{{\left(x_{1}-x_{2}\right)}^{2}+{\left(y_{1}-y_{2}\right)}^{2}+{\left(z_{1}-z_{2}\right)}^{2}}}{T}$$

where $\left(x_{1},y_{1},z_{1}\right)$ and $\left(x_{1},y_{1},z_{1}\right)$ are the coordinates of the RHEEL marker at the consecutive heel strike frames, and *T* is the time period between the two heel strikes.

An open-source musculoskeletal model, Freebody (v2.1) [28], was used for subsequent determination of internal forces based on the motion and kinetic data. The model’s predictions of tibiofemoral contact forces during gait have been validated using data from instrumented prostheses [29]. The processed static data were used for model calibration, scaling the measurements of gender-matched and height-approached subjects for whom the coordinates of bony landmarks, joint centers of rotation, and musculotendinous intersections were obtained using magnetic resonance imaging [29]. Knee moments were calculated by the operation of inverse dynamics, and knee angles were obtained through the rotation of the tibia local coordinate system (LCS) referenced to the femur LCS from the dynamic trial frame to the static trial frame. Next, the tibiofemoral forces in the tibia LCS were determined through static optimization in Freebody.

The obtained axial medial knee force (KMF) and knee moments (KAM and KFM) were expressed referenced to the subject’s body weight (BW) and the product of the body weight and height, respectively, to allow for comparisons between subjects. KAA was displayed and compared in degrees. The time frames were transformed to the percentage of stance with cubic spline interpolation.

The knee parameters considered in this paper included the KMF, KAM, KFM, and KAA. The evaluation of the pKMF with multiple predictors (pKAM, pKFM, pKAA, and WS) were completed by linear regression models in Matlab. Therefore, each dynamic trial produced one group of pKMF, pKAM, pKFM, pKAA, and WS. In total, there were 64 × 3 groups of these five variables for the three tasks. When exploring the regression models, pKAM or pKFM was first entered into the model as the sole predictor, then pKAM and pKFM were entered, next pKAM, pKFM, and pKAA, and last pKAM, pKFM, pKAA, and WS were entered into the model together. Finally, fifteen linear regression models were built for straight walking, step and crossover turning. The coefficient of determination, R^2^, was used to evaluate goodness of fit.

### 2.4. Statistical Analysis

In order to illustrate the difference of the considered parameters across different tasks, WS and the peaks of these parameters (pKMF, pKAM, pKFM, and pKAA) were obtained and compared with one-way ANOVA analysis. Additionally, the correlation coefficients of these parameters between any two of straight walk, step turn, and crossover turn were calculated and compared through one-way ANOVA analysis. Statistical procedures were performed using Matlab R2016b. An alpha level of 0.05 was set for all analysis.

## 3. Results

### 3.1. Walking Speed

The walking speeds of the three different walking tasks were 1.007 ± 0.078 ms^−1^, 0.975 ± 0.095 ms^−1^, and 0.970 ± 0.094 ms^−1^ for SW, ST, and CT, respectively. Statistical analysis demonstrated that there was no significant difference of walking speed among these tasks.

### 3.2. pKMF Evaluation with pKAM, pKFM, pKAA, and WS

The results of linear regression are listed in Table 1. The significance of the F-statistic (*p* < 0.001) indicated a good estimation of the relationship between the pKMF and the predictors using the linear regression model. For each task, the adjusted R^2^ increased with the number of the predictors, indicating more predictors were able to account for more of the variance of the pKMF. It should be noted that there was no significant model for ST and CT with the pKFM, as shown in Table 1.

**Figure 2:**
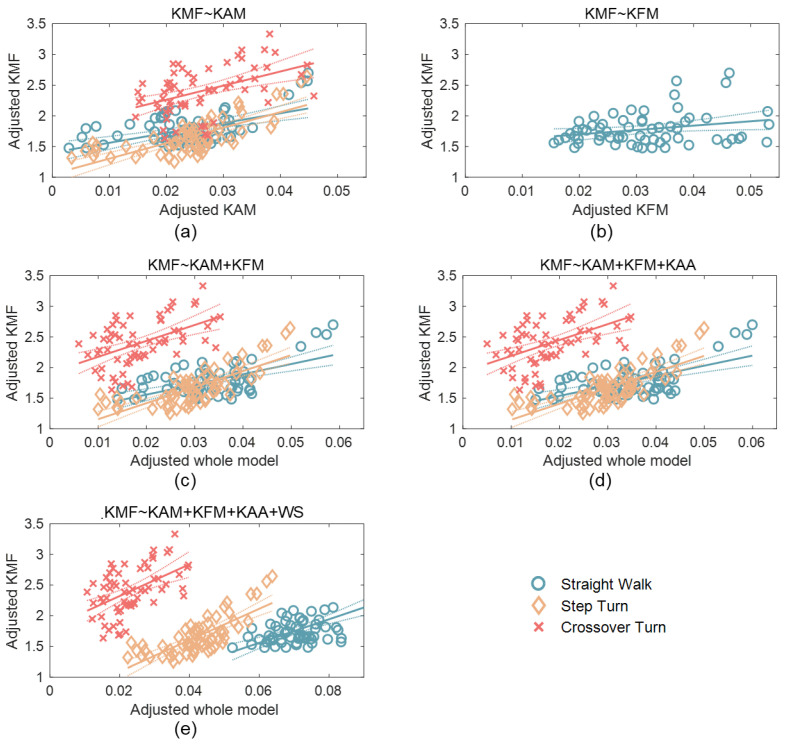
Scatter plots for the models considering (**a**) pKAM only; (**b**) pKFM only; (**c**) both pKAM and pKFM; (**d**) pKAM, pKFM, and pKAA together; and (**e**) pKAM, pKFM, pKAA, and WS. The solid lines: the fitted lines. The dot curves: 95% confidence bounds.

The models can be determined by the unstandardized coefficients listed in Table 1. The models for the three tasks with different predictors are plotted in Figure 2. Figure 2a,b are scatter plots of the pKMF with the pKAM and pKFM, respectively, as there is only one predictor in the models. The *x*-axes in Figure 2c–e indicate the predictors projected onto the best-fitting direction due to more than one predictor in the models. The models of SW and ST appear similar, with comparable coefficients. Additionally, the adjusted R^2^ values were also similar for these two tasks.

The model also demonstrated that the pKMF was significantly correlated with the pKAM and pKFM during ST, while only significantly correlated with the pKAM for ST and CT.

### 3.3. Knee Parameters of Different Level Walking Tasks

**Figure 3:**
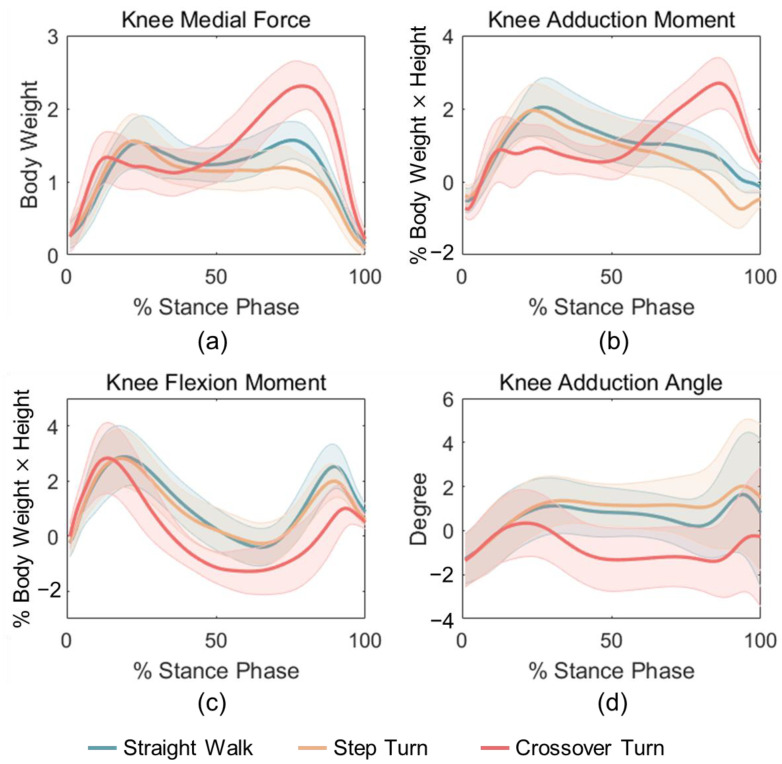
Mean curves of (**a**) pKMF, (**b**) pKAM, (**c**) pKFM, and (**d**) pKAA for SW, ST, and CT tasks.

The pKMF output by Freebody is shown in Figure 3a. The pKMF for crossover turning had an increased peak in the late stance, where the pKMF for step turning was smaller than that of straight walking. The calculated pKAM, pKFM, and pKAA of straight walk, step and crossover turn are shown in Figure 3b–d. The curve patterns of these variables during CT were markedly different from those of SW and ST, while the mean curves of the latter two tasks were relatively similar to each other. The peak KMF and KAM for SW and ST occurred in the early stance whereas that for CT in the late stance phase.

**Figure 4:**
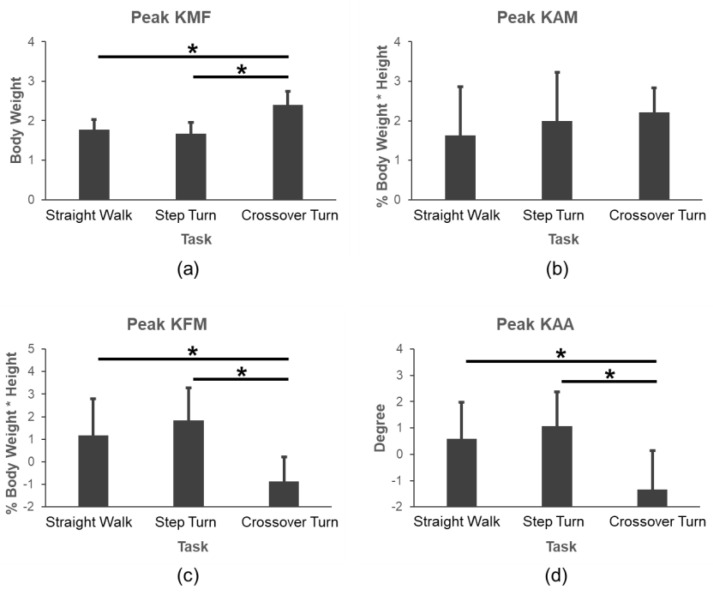
Comparisons of (**a**) pKMF, (**b**) pKAM, (**c**) pKFM, and (**d**) pKAA during SW, ST, and CT tasks. *: *p* < 0.001.

The comparison results of the pKMF, pKAM, pKFM, and pKAA under three level walking tasks is shown in Figure 4. It indicated that the pKMF increased, while the pKFM and pKAA decreased significantly during CT compared to those of SW and ST (*p* < 0.001), but there was no significant difference in the pKAM during different tasks.

**Figure 5:**
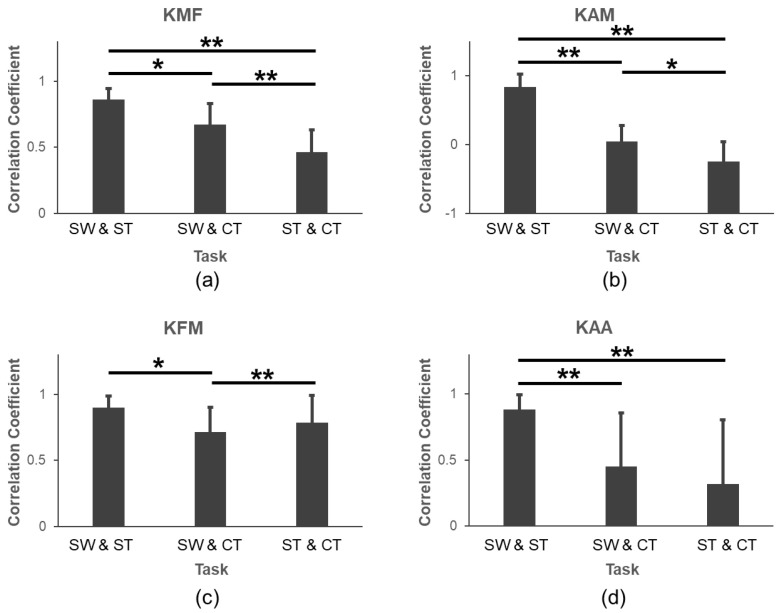
Comparisons of correlation coefficients for (**a**) pKMF, (**b**) pKAM, (**c**) pKFM, and (**d**) pKAA. *: *p* < 0.05; **: *p* < 0.001.

In Figure 5, we compared the correlation coefficients of various parameters under three difference tasks. For the pKMF, pKAM, and pKAA, the correlation coefficients between SW and ST were significantly larger than those between SW and CT (pKMF: *p* = 0.001; pKAM: *p* < 0.001; pKAA: *p* = 0.005) and those between ST and CT (pKMF: *p* < 0.001; pKAM: *p* < 0.001; pKAA: *p* < 0.001). For the pKFM, the correlation coefficient between SW and ST was significantly larger than that between SW and CT (*p* = 0.010).

## 4. Discussion

This study investigated the relationship between knee medial loading and some lower limb kinetic and kinematic variables, including the pKAM, pKFM, and pKAA for SW, ST, and CT. There have been a few studies focused on the relationship between the KMF and the KAM or KAA separately [15,30,31], and at most between the KMF and a combination of the KAM and KFM [17,19,22] during level walking, but there are no studies that consider all these variables together to evaluate the pKMF under various walking and turning tasks. Meanwhile, we provided the formulas to estimate the pKMF based on the pKAM, pKFM, and pKAA together and respectively, which can simplify the calculation of the pKMF and exhibit the relationship.

Our study showed that adding the pKAA to the linear regression models improved the pKMF evaluation with an increased determination of coefficient for SW and ST, but not appropriate for CT. In addition, this is the first study to compare the parameters and the pKMF regression models for three tasks (SW, ST, and CT). It can be deduced that SW and ST had a higher correlation, considering the knee joint parameters (Figure 5).

The three parameters (pKAM, pKFM, and pKAA) were in high accordance with those obtained by Taylor et al. [32], not only in curve shape, but also in the relation of the curves during these walking and turning tasks. Therefore, it seems reasonable to use these predictors to estimate the pKMF in this study. Previous studies in vivo reported that the maximal KMF was about 1.2–2.5 BW for gait [15,17,33], which was similar to 1.57 ± 0.25 BW during SW in our study. The KAM usually exhibits two peaks during the stance phase, with the second peak moment significantly smaller when walking with an increased foot progression angle [34]. The step turning here could be approximated as walking with a very large foot progression angle, and therefore, the KAM during the late stance of step turning was smaller than that of straight walking, as shown in Figure 3b.

The regression analysis revealed several interesting findings. Although it was shown that the pKFM was only significantly correlated with the pKMF during straight walking in Table 1, the addition of the pKFM during all three tasks could enlarge the R^2^ values of the models (pKAM:0.284 and 0.538 and 0.215 vs. pKAM and pKFM: 0.336 and 0.563 and 0.253). Furthermore, the models, for SW and ST, were better in estimating the relationship between pKMF and pKAM only or plus pKFM than CT (pKAM: 0.273 and 0.530 vs. 0.203; pKAM and pKFM: 0.314 and 0.548 vs. 0.228 in Table 1). Additionally, we found that the pKAA was not a significant predictor of the pKMF, and adding the pKAA did not improve the performance of the linear regression model in evaluating the pKMF. This result aligns with the findings of Adouni et al. [30], who also emphasized that the pKAA might not be as important. Therefore, we encourage use of the pKAM and pKFM when estimating the pKMF across different walking tasks.

Moreover, the models for SW and ST were similar models with approaching beta coefficients and fitting effect, whereas they were different from the models for CT. The pKMF value of CT was larger than that of SW and ST during the late stance phase (Figure 3). This seemingly was due to the shift in the center of gravity during the last phase of CT; however, this needs to be verified in future studies. Although the pKFM and pKMF have weak correlations in SW but not in ST and CT, adding the pKFM to the pKAM could increase the estimation of the pKMF (Table 1). According to Figure 4, we could know that the pKMF of CT is significantly higher than those in SW and ST, but the pKAA and pKFM were significantly lower. There was no significant difference in the pKAM during the three tasks. The correlation coefficients between SW and ST were significantly larger than those in SW and CT or ST and CT (Figure 5). This means that the kinematics and kinetics features of SW and ST were more similar. To be noted, the pKFM had the opposite effect on the pKMF for CT with a negative beta coefficient compared to SW and ST. The results can be interpreted as: the pKMF increased if the pKAM and pKFM both increased for SW and ST or if the pKAM increased for CT (Table 1). The absolute unstandardized coefficients for the pKAM were larger than that for the pKFM, meaning that any potential increase in the pKMF associated with an increase in the pKAM can be offset by a larger decrease in the pKFM during SW and ST. This is in accordance with previous results in [22].

Peaks of the KAM, KFM, and KAA were used to predict the peak of KMF in this paper. Due to the inconsistency of the peak time of each index, the coefficient of determination R^2^ of the models (in Table 1) were lower than previous studies, which had mostly selected the same time values of pKAM and/or pKFM corresponding to the pKMF for analysis [35]. However, such values of the KAM or KFM at the time of the peak KMF may not be the true peak values of the KAM and KFM. The method was not applicable; however, it had a higher correlation. Therefore, the peak value of each parameter was selected to establish the prediction models in this study. Furthermore, the KAM was undoubtedly the most important predictor with the largest absolute value of standardized coefficients, which verified that the KAM was the most effective and significant surrogate of the KMF [15]. The results gave an explanation about the phenomenon: a higher KAM implied larger KMF, which is believed to contribute to knee pain and cartilage defects and degeneration [36].

There were some limitations that we have to discuss. The most fundamental limitation was that the pKMF obtained by a previously validated musculoskeletal model does not represent the actual knee medial loading, which is impossible to measure in healthy subjects. Additionally, the moments of different joints may be correlated [37]. The moments of hip and ankle joints will be considered in future work. Finally, the evaluation of the pKMF was investigated among limited young healthy subjects. The results for elders and knee OA patients may be different; therefore, different types of subjects should be added for more accurate analysis. It should be noted that the aim of our study was not to provide a generalizable predictive regression equation for use by others. Whether the prediction model can be generalized for other subjects should be further validated. What can be generalized from our study is that the pKAM is a good surrogate parameter for different gait tasks (SW, ST, and CT), while the pKFM is only effective for SW.

## 5. Conclusions

This was the first study to simultaneously consider the relationship between pKMF and the pKAM, as well as the pKFM and pKAA, with some of the variables being significant predictors of KMF for SW, ST, and CT. The results showed that the pKAM and the pKFM were important predictors of pKMF, as the use of both the pKAM and the pKFM led to better estimations for these three levels of walking tasks. Additionally, the estimation models for SW and ST were found to be similar and more accurate than those for CT, possibly due to the different biomechanics involved in these tasks. Therefore, it is reasonable to consider the walking type when comparing substitute predictors of KMF, as different types of walking involve distinct biomechanics, leading to different estimation models.

## Figures and Tables

**Table 1:** Linear regression results for pKMF prediction. (Bold: *p* < 0.05).

Task	Predictors	Model	Beta Coefficients					R^2^ (Adj. R^2^)	Sig.
			Unstandardized	Std. Error	Standardized	t	Sig.		
Straight walk	pKAM	Const	1.402	0.081		17.268	**<0.001**	0.284 (0.273)	**<0.001**
		pKAM	16.042	3.232	0.533	4.963	**<0.001**		
	pKFM	Const	1.557	0.107		14.502	**<0.001**	0.071 (0.055)	**0.034**
		pKFM	7.090	3.269	0.266	2.169	**0.034**		
	pKAM pKFM	Const	1.222	0.115		10.731	**<0.001**	0.336 (0.314)	**<0.001**
		pKAM	15.545	3.154	0.517	4.941	**<0.001**		
		pKFM	6.092	2.830	0.228	2.181	**0.033**		
	pKAM pKFM pKAA	Const	1.207	0.121		9.958	**<0.001**	0.338 (0.305)	**<0.001**
		pKAM	15.185	3.297	0.505	4.606	**<0.001**		
		pKFM	6.348	2.886	0.238	2.200	**0.032**		
		pKAA	0.006	0.015	0.044	0.396	0.694		
	pKAM pKFM pKAA WS	Const	0.404	0.398		1.016	0.314	0.384 (0.343)	**<0.001**
		pKAM	19.126	3.710	0.636	5.156	**<0.001**		
		pKFM	4.645	3.583	0.062	0.459	0.648		
		pKAA	−0.005	0.016	−0.039	−0.340	0.735		
		WS	0.881	0.417	0.295	2.111	**0.039**		
Step turn	pKAM	Const	1.052	0.077		13.654	**<0.001**	0.538 (0.530)	**<0.001**
		pKAM	25.248	2.974	0.733	8.489	**<0.001**		
	pKAM pKFM	Const	0.894	0.113		7.896	**<0.001**	0.563 (0.548)	**<0.001**
		pKAM	25.671	2.925	0.746	8.778	**<0.001**		
		pKFM	5.183	2.764	0.159	1.875	0.066		
	pKAM pKFM pKAA	Const	0.885	0.119		7.471	**<0.001**	0.563 (0.541)	**<0.001**
		pKAM	25.572	2.972	0.743	8.604	**<0.001**		
		pKFM	5.251	2.798	0.161	1.877	0.065		
		pKAA	0.003	0.011	0.022	0.258	0.797		
	pKAM pKFM pKAA WS	Const	0.580	0.273		2.126	**0.038**	0.574 (0.545)	**<0.001**
		pKAM	25.399	2.962	0.738	8.574	**<0.001**		
		pKFM	2.758	3.435	0.085	0.803	0.425		
		pKAA	0.001	0.011	0.011	0.131	0.896		
		WS	0.395	0.318	0.130	1.240	0.220		
Crossover turn	pKAM	Const	1.803	0.152		11.825	**<0.001**	0.215 (0.203)	**<0.001**
		pKAM	23.002	5.576	0.464	4.125	**<0.001**		
	pKAM pKFM	Const	1.915	0.163		11.748	**<0.001**	0.253 (0.228)	**<0.001**
		pKAM	25.391	5.653	0.512	4.492	**<0.001**		
		pKFM	−5.623	3.211	−0.200	−1.751	0.085		
	pKAM pKFM pKAA	Const	1.929	0.170		11.328	**<0.001**	0.254 (0.217)	**<0.001**
		pKAM	25.342	5.697	0.521	4.448	**<0.001**		
		pKFM	−5.751	3.261	−0.204	−1.764	0.083		
		pKAA	−0.008	0.024	−0.035	−0.313	0.756		
	pKAM pKFM pKAA WS	Const	1.805	0.429		4.208	**<0.001**	0.255 (0.205)	**0.001**
		pKAM	25.167	5.767	0.508	4.364	**<0.001**		
		pKFM	−5.969	3.358	−0.212	−1.778	0.081		
		pKAA	−0.007	0.024	−0.035	−0.304	0.762		
		WS	0.442	0.442	0.037	0.315	0.754		

## Data Availability

The raw data supporting the conclusions of this article will be made available by the authors on request.
